# Determinants of time-to-recovery from hypertension by application of Weibull-Inverse Gaussian shared frailty model

**DOI:** 10.11604/pamj.2024.48.107.43082

**Published:** 2024-07-16

**Authors:** Yeshambel Kindu Yihuna, Nigist Mulu Takele, Essey Kebede Muluneh

**Affiliations:** 1 Department of Statistics, College of Natural and Computational Sciences, Debark University, Debark, Ethiopia,; 2 School of Public Health, Bahir Dar University, Bahir Dar, Ethiopia

**Keywords:** Hypertension, recovery, survival analysis, accelerated failure time, frailty model

## Abstract

**Introduction:**

hypertension is a major public health problem that is responsible for mortality. In Ethiopia, hypertension is becoming a double burden due to urbanization. The study aims to identify factors that affect the time to recovery from hypertension.

**Methods:**

in this study, a retrospective study design was used, and the data was collected in the patient´s chart from September 2016 to January 2018. Weibull-Inverse Gaussian shared frailty model was employed to identify factors associated with the recovery time of hypertension.

**Results:**

eighty-one percent of the sampled patients were recovered to a normal condition, and nineteen percent of the patients were censored. The median survival time for hypertensive patients to attain a normal condition was 13 months. Weibull-Inverse Gaussian shared frailty model was used for predicting the recovery time of hypertension patients. Unobserved heterogeneity in residences, as estimated by the Weibull-Inverse Gaussian shared frailty model, was θ = 0.385 and p-value = 0.00.

**Conclusion:**

age, systolic blood pressure, related disease, creatine, blood urea nitrogen, the interaction between blood urea nitrogen and age. Therefore, health-care providers give great attention, prioritize those identified factors and provide frequent counseling about reducing hypertension disease.

## Introduction

Hypertension is a chronic disease and the most common health problem resulting from high blood pressure during circulation. It is a silent public health trouble and a modifiable risk factor for non-communicable diseases. Globally, as the World Health Organization report shows, there have been 40 million deaths, and more than 70% of mortality was caused by non-communicable diseases in 2015 and 2016, respectively [1]. Annually, the number of worldwide deaths from noncommunicable diseases (NCDs) is expected to increase to 52 million by 2030 [2]. In the global world, the prevalence of hypertension is 1.3 billion individuals, which represents 31% of adults [3]. Among adults aged 18 years and older, the prevalence of hypertension was near 22% in 2014 (WHO) [2] and also hypertension is estimated to be the risk factor for 7.5 million deaths and 57 million disability individuals each year. High-income countries have a lower prevalence of hypertension (35%), compared to lower and middle-income countries (46%) [4].

Among total deaths due to cardiovascular disease, 80% occur in low- and middle-income countries, with the highest death rate reported in Africa [5]. This indicates that hypertension is one of the most commonly detected danger factors for cardiovascular disease in sub-Saharan African countries [6]. There were an estimated 74.7 million hypertension patients in sub-Saharan African countries, and by the year 2025, the estimated number of hypertension patients will be projected to be 125.5 million [7]. Ethiopia is one of the sub-Saharan African countries in which hypertension and its related risk factors are becoming a double burden due to urbanization and economic development. Hypertension is also one of the most serious non-communicable chronic diseases in Ethiopia [8]. In many developed countries, the prevention and control of hypertension have been given due attention. Though control of blood pressure and awareness about treatment are very limited in underdeveloped countries, including Ethiopia (WHO) [9].

Ethiopia has not methodically studied the epidemiological situation of hypertension, nor have they established systematic programs to create awareness, prevent, and control hypertension [10]. The capacity for controlling hypertension is very low; among ten patients, only eight are aware that their hypertension is not controlled. Hypertensive patients who have had a strong family history of hypertension, a higher heart rate, and a greater cardiovascular response controlled their blood pressure (BP) compared to the normotensive family history-negative control population [11]. In this study, the Weibull-Inverse Gaussian Shared Frailty Model was used to analyze correlated hypertension by assuming that patients within the same cluster (residence) share similar risk factors. The aim of this study is to determine the time to recover from hypertension and identify significant predictors at Felege Hiwot Referral Hospital (FHRH).

## Methods

**Sample design:** this study used a retrospective study design. The study was conducted at Felege Hiwot Referral Hospital, which provides good services for the management of hypertension in the Bahir Dar Town administration. Bahir Dar is found at a distance of 565 kilometers from Addis Ababa and is located on the southern shore of Lake Tana, the source of the Blue Nile. According to the report of the chief executive officer of FHRH, the total population served by the hospital is about 12 million per year, and there are around 400 healthcare professionals. This study used retrospective survival data and secondary data that were found at Felege Hiwot Referral Hospital. The patients with incomplete recordings of baseline data were excluded from the study.

### Variables

**Outcome variable:** the response variable of this study is the survival time of the hypertensive patients, which is the length of time from the start date of taking antihypertensive drugs until the date of recovery (becoming a normal condition of hypertension or censored, measured in months).

**Independent variables:** several predictors were considered in this study to investigate the determinant factors of time to recover from hypertension. The candidate predictors included in this study were sex, age, residence, systolic blood pressure (mmhg), creatine (mg/dl), other related diseases, blood urea nitrogen (mg/dl), diastolic blood pressure (mmhg), and a number of medications.

**Data source:** the source of the population consists of all hypertensive patients who were joined for follow-up in Felege Hiwot Referral Hospital from September 2016 to January 2018.

**Inclusion criterion:** all hypertension patients whose ages were greater than or equal to 18 years and who were starting hypertension treatment at the Felege Hiwot Referral Hospital from September 2016 to January 2018 were included in this study.

**Exclusion criterion:** hypertension patients whose age is less than 18 years at Felege Hiwot Referral Hospital.

**Sample size determination:** in survival analysis, the sample size is determined using the sample size determination formula [12]. In this study, α = 0.05 and β = 0.1 were used to determine the sample size. From the pilot survey estimate of survivor function for standard treatment, the median survivor time was 16 months, and the survival rates at seventeen, thirty-two, and forty-seven months were 0.414, 0.265, and 0, respectively. The new treatment is expected to increase the survival rate at sixteen months from 0.69 under the standard treatment to 0.79. Subsequently, in this study, patients were recruited over a 30-month accrual period, and there is to be a follow-up period of 17 months. Based on the above results, the sample size is given as 299.

**Statistical methods:** in this study, the Weibull-Inverse Gaussian Shared Frailty Model was applied to analyze the hypertension data set. In order to compare groups of subjects, this study used Kaplan-Meier´s plots [13]. The Accelerated Failure Time model (AFT), which makes inferences about the potential risk of observation on the timing of events with the equation defined by Kleinbaum and Klein [14] formulated as:

*S* (*t/x*) *= S0* {t * exp (*ȧ x*)}

Where S(t/x) is the conditional survival function represents the probability that a survival time T exceeds t, given a specific set of covariates or predictors, X, equal to x. S0(t) is the baseline survival function, which represents the survival probability at time t when all covariates x is zero or at their reference level. Exp (α´x) is the exponential function of a linear combination of covariates x, where α is a vector of coefficients.

Here, the researchers consider the log scale of the AFT model with respect to the time given analogous to the classical linear regression approach. In this approach, the natural logarithm of the survival time, Y = log (T), is employed. This is the natural transformation made in linear models to convert positive variables to observations on the entire real line. A linear model is assumed for Y: Y=log(T)=μ+α’x+δε. Where α’ = (α1, α2... αp) is a vector of regression coefficients, μ = intercept, δ = is the scale parameter and, ε = is the error distribution assumed to have a particular parametric distribution.

Statistical models and methods proposed to model failure time data assume that the observations are statistically independent of each other. However, this does not hold in many applications. The concept of frailty provides a suitable way to introduce random effects in the model to account for association and unobserved heterogeneity. In a parametric model, estimation is then conducted by maximizing the marginal log-likelihood [15]. In the study, Akaike information criteria (AIC) and Bayesian information criterion (BIC) criteria were used to compare various candidate models, and the model with the smallest value was considered a better fit. The response variable for this study was the recovery time of hypertension patients after taking antihypertensive drugs, which is recorded until 1 if an event occurs and survives. The censoring indicator is 0 for censored observations and 1 for events that occurred in this case.

## Results

**Descriptive statistics:** random samples of 299 hypertensive patients were included in this study. Of these, 242 (81%) were recovered and 57 (19%) were censored observations during the follow-up period. Among those sampled patients, 173 (57.9%) were female and 175 (58.5%) came from urban areas. Furthermore, 60 (20%) of the patients had diabetes, 58 (19.5%) had kidney disease, 36 (12.0%) had other disease and 145 (48.5%) had no any disease (Table 1).

**Table 1:** demographic characteristics of time to recovery from hypertension patients at Felege Hiwot Referral Hospital

Factors	Categories	Status		
		Censored (%)	Event (%)	Total (%)
Sex	Male	12(4.0)	144(38.0)	156(42.0)
	Female	45(15.2)	128(42.8)	173(58.0)
Residence	Rural	18(6.0)	106(35.5)	124(41.5)
	Urban	39(13.0)	136(45.5)	175(58.5)
Creantine (mg/dL)	≤1.5	18(6.0)	112(37.5)	130(43.5)
	>1.5	39(13.0)	130(43.5)	169(56.5)
Other related disease	Diabetes	28(9.4)	32(10.7)	60(20.0)
	Kidney	18 (6.0)	40(13.5)	58(19.5)
	Other	4 (1.3)	32(10.7)	36(12.0)
	None	7(2.3)	138(46.2)	145(48.5)
Blood urea nitrogen (mg/dL)	≤35	14(4.7)	120(40.1)	134(44.8)
	>35	43(14.4)	122(40.8)	165(55.2)
Number of medications	One	53(17.7)	177(59.2)	230(76.9)
	Two or more	4 (1.4)	65(21.7)	69(23.1)

**Figure 1:**
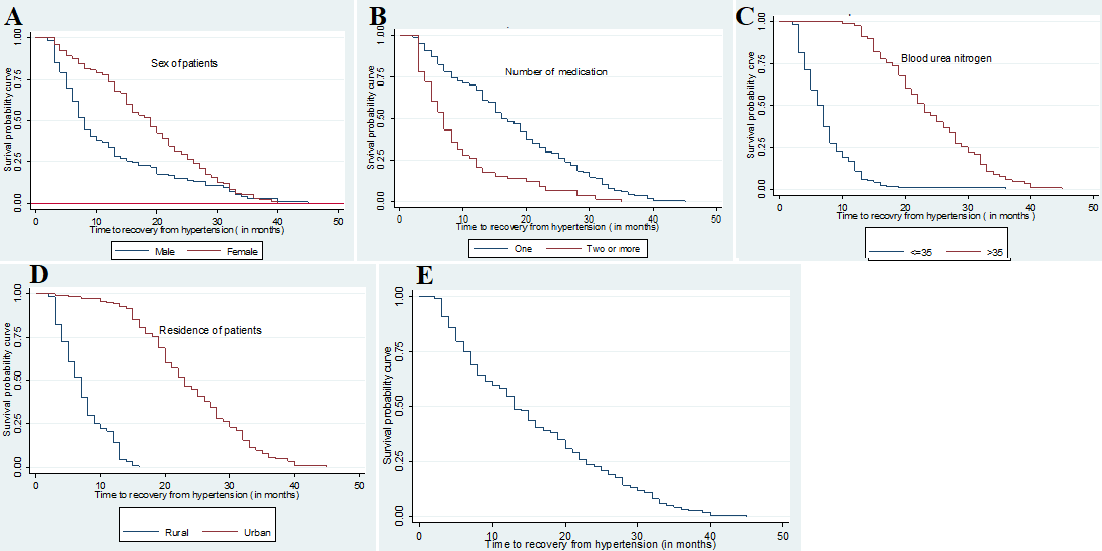
A,B,C,D) survival probability curves of time to recovery from hypertension (in months) versus different categories of socioeconomic factors; E) overall survival probability curves of time to recovery from hypertension (in months)

**Kaplan-Meier (KM) survival curves for different groups:** non-parametric survival analysis is very important to visualize the survival time-to-recovery of patients from hypertension at FHRH under different levels of covariates. Moreover, it gives information on the shape of the survival of the hypertension data set. The resulting KM survival curve based on the FHRH dataset is shown (Figure 1 (A,B,C,D)). we can observe the survival distribution of survival time of hypertension patients by sex. The curve of female patients lying above as compared to male patients indicates that male patients attained good control of hypertension which is faster than female patients. This means the probability of prolonging recovery time at a given time for female patients is greater as compared to male patients. The survival of the patients who were using one medication is greater as compared to the survival of the patients who were used two or more medications. That is, the probability of prolonging recovery time at a given time for patients who received one medication is greater as compared to those who received two or more medication group. The curve of patients living in urban area lying above as compared to patients live in rural area indicates that patients living in urban area attained good control of hypertension which is faster than patients live in rural area. This means the probability of prolonging recovering time for patients living in urban area is greater as compared to patients live in rural area and the graph clearly states that there is heterogeneity in recovery time of hypertension among residents. The overall plot of the KM curves to the survival of time-to-recovery from hypertension is shown (Figure 1E). The survival plot decreases at increasing rate at the beginning and decreases at decreasing rate latte. The plot of the KM curves reflects that the probability of survival decreases when the recovery time is increased. The results of both log-rank and Breslow test for survival differences were highly significant. By using both tests, there were significant differences in survival experience among groups of sex, residence, related disease, creatine, blood urea nitrogen, and a number of medications. These showed that all categorical variables had statistically significant differences in survival probabilities (Table 2).

**Table 2:** results of log-rank and Wilcoxon test for each categorical variable of hypertension

		Log-rank test		Wilcoxon (Breslow)	
Covariates/factors	DF	Chi-square	p-value	Chi-square	p-value
Sex	1	13.83	0.002	33.67	0.000
Residence	1	255.93	0.000	216.56	0.000
Related diseases	3	256.74	0.000	188.52	0.000
Creantine	1	82.26	0.000	81.06	0.000
BUN	1	222.29	0.000	209.71	0.000
Number of medications	1	40.75	0.000	46.69	0.000

DF: degree of freedom; P-value: the probability of observing a test statistic; BUN: blood urea nitrogen

**Statistical analysis:** the researcher fitted the data using the Weibull-Inverse Gaussian shared frailty model. A univariable analysis was performed for each potential model to see the effect of each predictor on the survival time-to-recovery of patients from hypertension and to select predictors to be included in the multivariable analysis. From the result in univariable analysis, age, sex, systolic blood pressure, diastolic blood pressure, related disease, creatine, blood urea nitrogen and a number of medications were significant at 25% level of significance in all baseline distributions. Hence, based on the univariable analysis, these variables were included in the multivariable analysis.

The multivariable analysis indicates that age, related disease, creatine, blood urea nitrogen, and the interaction between age and blood urea nitrogen were significant at p <0.05. This study checked the heterogeneity using residence as a frailty term and investigated risk factors associated with the recovery time of hypertension patients. The variance of the frailty was significant in inverse Gaussian shared frailty distribution with Weibull baseline distribution at p <0.05. This shows the presence of heterogeneity and necessitates the frailty models. The variance parameter θ in a shared frailty model indicates a measure of the degree of correlation and provides information on the variability in the population of clusters. This indicated that the Weibull-Inverse Gaussian shared frailty model described the hypertension dataset with the value of variance parameter θ was 0.385. The result of the Weibull-Inverse Gaussian shared frailty model. From this result, the frailty term θ=0.385 indicates that there is a presence of heterogeneity between residences. A likelihood ratio test for the hypothesis θ=0 is shown in the table below indicating a chi-square value of 21.48 with one degree of freedom resulting in a highly significant with p <0.05. This implied that the frailty component had significant contribution to the model. Kendall's tau (τ) is a tatistic used to measure the association within the residence. From the results of this study, the value of Kendall's tau (τ) for the Weibull-Inverse Gaussian frailty was 0.141. The estimated value of the shape parameter of Weibull-Inverse Gaussian frailty was (p=3.803). This indicates that the shape of hazard functions is increasing up as time increases since its value is greater than one.

Weibull-Inverse Gaussian shared frailty model results (Table 3) control for other variables: acceleration factor, 95% CI, and p-value of the variable systolic blood pressure were 1.002, (1.00, 1.004) and 0.022 respectively. These indicated that systolic blood pressure was significant factors for the recovery time and for a one mmHg increases in systolic blood pressure the recovery time of hypertensive patients were prolonged by 1.002 times in month. Acceleration factor and its 95% CI for related disease category of kidney, others and none were (φ =0.859, 95% CI=0.760, 0.971), (φ= 0.628, 95% CI=0.550, 0.718) and (φ=0.557, 95% CI=0.478, 0.649) respectively. This showed that patients having kidney disease, others and who have no related disease shortened the recovery time by a factor of 0.859, 0.628, and 0.557 respectively compared to diabetes patients (reference). The 95% CI did not include one that showed that related disease was a significantly important factor for the recovery time at p <0.05.

**Table 3:** multivariable result of Weibull Accelerated Failure Time model for the recovery time of hypertension patients

Covariates	Categories	Coef	Ф	95%CI		SE(β)	P-value
				Lower	Upper		
Age		0.013	1.013	1.009	1.017	0.002	0.000
Systolic blood pressure		0.002	1.002	1.00	1.004	0.001	0.022
Related disease	Diabetes	Ref	1				
	Kidney	-0.152	0.859	0.759	0.971	0.063	0.015
	Other	-0.465	0.628	0.549	0.718	0.068	0.000
	None	-0.586	0.557	0.478	0.649	0.078	0.000
Creatine	≤1.5	Ref	1				
	>1.5	0.133	1.142	1.051	1.241	0.042	0.002
Blood urea nitrogen	≤35	Ref	1				
	>35	0.968	2.632	1.97	3.517	0.148	0.000
Blood urea nitrogen with age	≤35	Ref	1				
	>35	-0.012	0.988	0.983	0.993	0.002	0.000

ϴ=0.385; τ= 0.141; P=3.803; λ=0.001; AIC=151.08; likelihood-ratio test of theta=0: chibar 2(01) = 21.48; prob ≥chibar2 = 0.000; Coef=coefficient; Ф =acceleration factor; CI =confidence interval; SE( β) = standard error of the coefficient; ϴ = variance of the random effect; τ = Kendall´s tau p =shape parameter; λ=scale parameter; AIC= Akaike information criteria; prob=probability; chibar2= Chi-square

The acceleration factor and its 95% CI for the variable creatine were 1.142 and (1.051, 1.241) respectively. These indicated that creatine was a significant factor for the recovery time and showed that the recovery time of patients, who have had creatine levels >1.5 mg/dL, was prolonged by 1.142 times as compared to patients who have had creatine levels ≤1.5 mg/dL. The interaction between blood urea nitrogen and age had a statistically significant effect on the recovery time, suggesting that as age increased by one year, the acceleration factor for patients who have had blood urea nitrogen >35 mg/dL was prolonged by exp (0.013-0.012) =1.001 with p <0.05 as compared to those patients who have had blood urea nitrogen ≤35 mg/dL.

## Discussion

Age, sex, residence, systolic blood pressure, diastolic blood pressure, related disease, creatine, blood urea nitrogen, and the number of medications affect the time to recovery from hypertension. Both univariable and multivariable analyses were performed to examine factors that affect the recovery time of hypertension patients. The univariable analysis revealed that variables age, sex, systolic blood pressure (SBP), diastolic blood pressure (DBP), related disease, creatine, blood urea nitrogen (BUN), and number of medications were significant at a 25% level of significance. All significant covariates in univariable analysis were included in multivariable analysis. This research showed that there was a clustering (frailty) effect on recovery time of hypertension patients which might be due to the heterogeneity in residence. Then, patients coming from the same residence share similar risk factors related to recovery time, and indicating that it was important to consider the cluster effect.

The finding of this study showed that systolic blood pressure had a positive significant effect on recovery time that is as systolic blood pressure increases the recovery time of hypertension patients is also prolonged. The finding was consistent with Xiong *et al*. and Workie *et al*. [16,17]. Related disease is another prognostic factor that significantly predicts the recovery time of patients with hypertension. It showed that patients having kidney disease, other and have no related disease shortened the recovery time compared to diabetes patients; this study is supported by the study [18].

Another potential risk factor that accelerates the recovery status of patients from hypertension is creatine and showed that the recovery time of hypertensive patients, who have had creatine >1.5 mg/dL, was prolonged. This study was consisting with Workie *et al*. and Angaw *et al*. [17,19]. The results of this study also suggested that the interaction between blood urea nitrogen and age had a statistically significant effect on the recovery time, suggesting that as age increases, the acceleration factor for patients who have had blood urea nitrogen level >35 mg/dL was prolonged as compared to those who have had blood urea nitrogen ≤35 mg/dL. The related literature has not been done by using the interaction effect of variables in the area of hypertension. Consequently, this study is unique in that the interaction effect of variables was assumed to see whether the effect of one variable is different or not on the acceleration factor of another variable. In the course of doing this, the time of recovery for patients with hypertension did not depend on sex which is similar to the study [20] suggested that sex was not statistically associated with hypertension using logistic regression model in Northwest Ethiopia, Addis Ababa and Zimbabwe respectively.

**Study limitation:** for some observations, predictors were not registered as the baseline. Due to these observations, predictors not registered at the start of treatment were ignored from the study. This might mislead the findings of the study. Convergence problem for analyzing Inverse Gaussian shared frailty model with log-logistic baseline distribution.

## Conclusion

The aim of this study is to identify factors that affect time-to-recovery from hypertension patients using survival models at FHRH, Bahir Dar. The Weibull-Inverse Gaussian shared frailty model was fitted model for the recovery time of patients from hypertension. There was a frailty (clustering) effect on time-to-recovery from hypertension that arises due to differences in the distribution of the timing of recovery among residents in Felege Hiwot Referral Hospital. This indicates that there is the presence of heterogeneity and necessitates the frailty models. The result of the Weibull-Inverse Gaussian shared frailty model showed that systolic blood pressure, related disease, creatine, and interaction between blood urea nitrogen and age were found significant predictors for the recovery time of patients among patients with hypertension in Felege Hiwot Referral Hospital. Among these significant predictors, systolic blood pressure and creatine prolong the timing of recovery while related disease and the interaction between blood urea nitrogen and age shorten the timing of recovery for patients with hypertension. On the other hand, sex, diastolic blood pressure, and number of medications were not significant predictors for the recovery time of patients with hypertension.

### What is known about this topic

- *A frailty model provides a suitable way to introduce random effects in the model to account for association and unobserved heterogeneity that modifies multiplicatively the hazard function of an individual;*
- *The median survival time of hypertensive patients to attain normal condition is long*.

### What this study adds

- *Residence has significant variation in time-to-recovery from hypertension;*
- *Since hypertension patients in the same residence bear a resemblance to more than patients in other residences clustering among residences was considered the clustering effect and Weibull-Inverse Gaussian shared frailty model was used to model the data*.
